# Evaluation of a 3A-truncated foot-and-mouth disease virus in pigs for its potential as a marker vaccine

**DOI:** 10.1186/1297-9716-45-51

**Published:** 2014-05-01

**Authors:** Pinghua Li, Zengjun Lu, Xingwen Bai, Dong Li, Pu Sun, Huifang Bao, Yuanfang Fu, Yimei Cao, Yingli Chen, Baoxia Xie, Hong Yin, Zaixin Liu

**Affiliations:** 1 State Key Laboratory of Veterinary Etiological Biology, National Foot and Mouth Disease Reference Laboratory, Key Laboratory of Animal Virology of Ministry of Agriculture, Lanzhou Veterinary Research Institute, Chinese Academy of Agricultural Sciences, No. 1 Xujiaping, Yanchangbao, Lanzhou, Gansu 730046, PR China

## Abstract

Foot-and-mouth disease (FMD) is a highly contagious and economically devastating disease of cloven-hoofed animals in the world. The disease can be effectively controlled by vaccination of susceptible animals with the conventional inactivated vaccine. However, one major concern of the inactivated FMD virus (FMDV) vaccine is that it does not allow serological discrimination between infected and vaccinated animals, and therefore interferes with serologic surveillance and the epidemiology of disease. A marker vaccine has proven to be of great value in disease eradication and control programs. In this study, we constructed a marker FMDV containing a deletion of residues 93 to 143 in the nonstructural protein 3A using a recently developed FMDV infectious cDNA clone. The marker virus, r-HN/3A_93–143_, had similar growth kinetics as the wild type virus in culture cell and caused a symptomatic infection in pigs. Pigs immunized with chemically inactivated marker vaccine were fully protected from the wild type virus challenge, and the potency of this marker vaccine was 10 PD_50_ (50% pig protective dose) per dose, indicating it could be an efficacious vaccine against FMDV. In addition, we developed a blocking ELISA targeted to the deleted epitope that could clearly differentiate animals infected with the marker virus from those infected with the wild type virus. These results indicate that a marker FMDV vaccine can be potentially developed by deleting an immunodominant epitope in NSP 3A.

## Introduction

Foot-and-mouth disease (FMD) is a highly contagious disease of cloven-hoofed animals, including cattle, pigs, goats, sheep, and other species of wild ruminants, that is characterized by the appearance of vesicles on the feet and mouth. In endemic countries, FMD causes severe economic loss as a result of a decline in productivity, costs of control measures, and international trade restrictions imposed on livestock and animal products, making FMD the most economically important disease of livestock worldwide. In the past, FMD outbreaks have occurred in most areas of the world, with the exception of Greenland, Iceland, New Zealand, and the smaller islands of Oceania [1]. Currently, FMD virus (FMDV) is enzootic in all continents except Australia and North America.

The causative agent, FMDV, belongs to the genus *Aphthovirus* in the family *Picornaviridae*, and exists as seven antigenically and genetically distinct serotypes: O, A, C, Asia1, SAT-1, SAT-2, and SAT-3. The virus is a non-enveloped particle of icosahedral symmetry containing a single-stranded, positive-sense RNA genome approximately 8.5 kb in length. The viral genome has a single open reading frame (ORF) that is translated into a polyprotein, which is processed by virus-encoding proteases (L, 3C, and 2A) to yield four structural (1A, 1B, 1C, and 1D) and ten nonstructural proteins (NSP) (L, 2A, 2B, 2C, 3A, 3B1-3, 3C, and 3D), as well as some relatively stable precursor proteins. Although the mature NSPs, as well as some of their protein precursors are involved in viral RNA replication, their exact role in the viral life cycle is still not fully understood [2,3].

In endemic regions, FMD control has been largely based on regular vaccination with whole–virus inactivated vaccines, slaughter of infected and in-contact animals, together with limitation of movement of susceptible animals and animal products. Although inactivated FMD vaccines have been used for many years and proved quite effective in control of clinical diseases [4], vaccinated animals cannot be serologically distinguished from those infected with the wild type (WT) virus, which is very important in disease control and eradication programs. To date, the application of 3ABC NSP-based strategies for differentiating infected from vaccinated animals (DIVA) have been successfully implemented to identify infection in vaccinated populations, but there is still some concern of asymptomatic virus carriers without positive reaction in NSP serological tests [5,6]. Of particular concern is the fact that some vaccine formulations may have residual NSPs, which makes it difficult to identify infection in repeatedly-vaccinated populations [7,8]. Therefore, there is an increased need for development of improved vaccines with a DIVA property against FMDV.

A marker vaccine, also called the DIVA vaccine, enables accurate serological differentiation between infected and vaccinated animals in conjunction with a companion serological test, and has proved extremely useful in outbreak control and sera-surveillance of infectious diseases. It has been shown that the vaccines with DIVA properties have successfully been used to control and eradicate some infectious animal diseases [9-11]. In addition, extensive studies have also demonstrated that the marker vaccines combined with accompanying diagnostic methods make serosurveillance of infectious diseases possible in endemic areas where a vaccination program has been or is being implemented [11-13]. In the present study, we describe the generation of a marker FMDV (r-HN/3A_93–143_) containing a deletion of residues 93 to 143 (this region harbors an immunodominant B-cell epitope of residues 99 to 105) in 3A protein using a recently developed FMDV full-length infectious cDNA clone [14]. The marker virus exhibited similar growth kinetics to WT virus in culture cell, and caused a symptomatic infection in pigs. The marker vaccine prepared from binary ethylenimine (BEI)-inactivated r-HN/3A_93–143_ proved to be effective in protecting pigs from the WT virus challenge. Furthermore, a developed blocking ELISA targeted to the deleted epitope could clearly differentiate animals infected with the marker virus from those infected with the WT virus.

## Materials and methods

### Cells, viruses and antibodies

BHK-21 and BSR/T7 cells [15] were propagated as described previously [16]. BSR/T7 cells were used to recover the recombinant virus and BHK-21 cells were used for titration, in vitro growth, and plaque assays.

WT FMDV r-HN (referred to here only), derived from a plasmid encoding the complete FMDV O/HN/CHA/93 genome (pOFS) [14], was passaged four times in BHK cells to further experiments.

MAb 3A24 directed against AEKNPLE (residues 99–105) epitope in NSP 3A of FMDV and MAb 3B4B1 directed against GPYAGPMER (residues 1–9) epitope in NSP 3B2 were obtained from the Lanzhou Veterinary Research Institute (LVRI). The region “AEKNPLE” was proven to be an immunodominant B-epitope in 3A protein by detecting the ability of FMDV reference sera recognized this region using a peptide ELISA in our lab (data not yet published). Fluorescein isothiocyanate (FITC)-conjugated goat anti-mouse IgG antibody was purchased from Sigma.

### Construction and rescue of the marker FMDV

A full-length infectious cDNA clone (pOFS) of the FMDV O/HN/CHA/93 was used to engineer the targeted deletion in 3A protein via an overlap extension method as previously described [17]. Briefly, two flanking fragments (fragment A and B) were PCR amplified from pOFS by primer sets HN-1 F/3A93-143R and 3A93-143 F/HN-4R. After amplification of the flanking regions, the two amplicons were mixed and subjected to the overlapping extension PCR with external primer pair HN-1 F/HN-4R. The overlapping genome fragment was digested with *Bgl* II and *Nru* I, and then cloned between the *Bgl* II and *Nru* I sites of pOFS to construct the final mutant full-length clone. After construction, the PCR-amplified region was sequenced for verification of the introduced deletion. The primers used for construction of the deletion mutant were listed in Table 1.

**Table 1:** Primers used for the construction of the mutant FMDV full-length cDNA clone with the targeted deletion in NSP 3A

**Primer**	**Sequence(5′ → 3′)**	**Usage**
HN-1 F	CAAGAAGTGATTGAGCGGGT	Amplification of fragment A
3A93-143R	GGTTGTTCCCTCCCGGGCACGTCATCCAGTGAGTCATCCA	
3A93-143 F	TGGATGACTCACTGGATGACGTGCCCGGGAGGGAACAACC	Amplification of fragment B
HN-4R	GTTCCCTTCTTCATTCTCGC	

The *Not* I-linearized mutant construct was transfected into BSR/T7 cells using Lipofectamine™ 2000 (Invitrogen, Carlsbad, CA, USA) as described previously [15]. After transfection, cells were monitored until a typical cytopathic effect of FMDV became apparent. Virus recovered from the transfected supernatant was passaged up to four times in BHK-21 cell monolayers, and the complete viral genomes of the marker virus, designated r-HN/3A_93–143_, as well as WT virus of passage 4 [16] were confirmed by nucleotide sequencing. The virus stocks collected at passage 4 were used for growth studies, plaque assays, animal experiments, and production of inactivated vaccines. Virus titer was determined by calculating the 50% tissue culture infectious dose per mL (TCID_50_/mL).

### Detection of progeny viral RNA

To analyze the stability of the deletion in 3A protein of FMDV, the marker virus was further passaged for 9 rounds (p13) in BHK-21 cells, and total viral RNAs were extracted from the supernatants of each passage using a QIAamp Viral RNA Mini kit (Qiagen, Valencia, CA, USA) according to the manufacturer’s protocol. One-step RT-PCR was performed with the HN-1 F/HN-4R primer pair. The PCR products were then purified and sequenced directly to confirm the presence of the targeted deletion in the marker virus.

### Analysis of growth of the marker FMDV

BHK-21 cell monolayers in 6-well plates were infected either with r-HN or r-HN/3A_93–143_ at an multiplicity of infection (MOI) of 1 and incubated at 37 °C in 5% CO_2_. After 1 h adsorption, the inoculum was removed, and cell monolayers were washed twice with serum-free minimal essential medium (MEM) to remove unattached viruses. After washing, 2 mL complete medium was added, and the plates were further incubated at 37 °C. The virus-infected supernatants were collected at different time points (0, 4, 8, 12 and 24 h post-infection), and the virus titer was determined by TCID_50_ as described above. The marker virus and WT virus were characterized by plaque assay in BHK-21 cells. Plaques were visualized under a gum tragacanth overlay stained at 48 h post-infection as previously described [18].

### Immunofluorescent assay

BHK-21 cells (2 × 10^5^) grown on a six-well plate were either mock infected or infected with r-HN or r-HN/3A_93–143_ at an MOI of 1. After 6 h post-infection, the cells were fixed with 4% paraformaldehyde for 20 min at room temperature, permeabilized for 20 min with 0.5% Triton X-100 in PBS, and blocked for 1 h with 10% bovine serum albumin in PBS. Then, the cells were incubated for 1 h with MAb 3A24 or 3B4B1, and then stained with fluorescein isothiocyanate (FITC)-conjugated goat anti-mouse IgG antibody for another 1 h. The cells were examined in an Olympus BX40 inverted fluorescence microscope.

### Western blot

The cell lysates from r-HN or r-HN/3A_93–143_ infected BHK-21 cells were prepared and separated on a 15% polyacrylamide gel. The resolved proteins were transferred to PVDF membranes by standard methods. Blots were blocked for 1 h in 5% nonfat milk powder in PBS (pH 7.4), and the membranes were reacted with MAb 3A24 or 3B4B1. After reaction, the membranes were incubated with horseradish peroxidase (HRP)-conjugated goat anti-mouse IgG antibody. Visualization of detected proteins was achieved using diaminobenzidine (DAB).

### Animal experiments

Animal experiments were performed under Biosafety Level 3 conditions in the animal facilities at LVRI following the protocol approved by the Review Board of LVRI, Chinese Academy of Agricultural Sciences (Permission number:SYXK-GAN-2004-0005). At the beginning of the experiments, all animals were negative for FMDV-specific antibodies. The animals were euthanized by intravenous injection of sodium pentobarbital at the end of all experiments.

FMDV O/HN/CHA/93 isolate contains a deletion of residues 93 to 102 in 3A protein, which is responsible for the virus’s inability to cause disease in cattle [16,19]. Therefore, in this study, we investigated the virulence of the marker virus in swine. Briefly, six 3-month-old pigs were divided into two groups (*n* = 3 per group), and each group was kept in a separate room. Group 1 was inoculated with the WT virus, and group 2 was inoculated with the marker virus. Inoculation was performed in the heel bulb, with each pig receiving approximately 10^6^ TCID_50_ in a volume of 0.2 mL. All animals were monitored daily for signs of FMD infection. Lesion scores were scored as previously described [20]. Briefly, clinical signs were based on affected sites that were clearly distinct from inoculation sites, and were scored using the following criteria: mouth, nostril, or tongue lesion beyond inoculation site = 1; one or more lesions per foot = 1. The maximum score is 5. Blood samples were collected from the inoculated animals on the following days post-infection (dpi) 0, 1, 2, 3, 4, 5, and 7, and FMDV RNA in blood were detected by rRT-PCR as previously described [21]. Furthermore, serum samples collected at 7, 14, 21, and 28 dpi were tested for FMDV-specific antibodies as previously described [22]. The antibodies to NSP 3ABC were also detected at 28 dpi by an FMDV NSP 3ABC-I-ELISA kit from LVRI [23]. Viral RNAs were extracted from vesicle samples, reverse transcribed, and sequenced.

### Vaccination and challenge of swine

The r-HN/3A_93–143_ and r-HN vaccine antigens were harvested from supernatants and lysates of infected BHK-21 cell cultures (a total of 4 × 10^8^ cells), and inactivated with BEI for 24 h at 25 °C. The vaccine antigens were concentrated by polyethylene glycol precipitation and purified through 10-30% sucrose density gradients as described previously [24]. Two vaccines were prepared as water-in-oil-in-water emulsions with Montadine ISA 201 (Seppic, Paris, France). The antigen concentration present in the experimental vaccines was then estimated using a previously described method [24]. Ten FMD-seronegative pigs (3 months of age) were randomly separated into two groups of four animals each, and one control group of two animals. Subsequent to an initial acclimatization period, the pigs were vaccinated intramuscularly with 2 mL (2 μg per 2 mL) chemically inactivated r-HN/3A_93–143_ and r-HN vaccine, respectively. Control animals were inoculated with 2 mL MEM. Four-weeks post-vaccination, all animals were rebled, and sera were tested for the presence of FMDV specific antibodies and 3ABC antibodies. At 28 days post-vaccination (dpv), all pigs were challenged intramuscularly in the neck with 1000 ID_50_ (50% pig infectious doses) of the WT virus. This challenge model of FMDV has been well developed for the FMD vaccine potency test in LVRI [25], and also has been used as a standard method for detecting potency of all commercially inactivated vaccines for pigs in China [26]. The animals were examined daily for fever and clinical signs. On 21 days post-challenge, serum samples obtained from all experimental pigs were tested for the presence of antibodies to 3ABC.

### Assessment of potency of the marker vaccine in swine

The potency of the marker vaccine was estimated in vaccinated pigs. The protocol was similar to the cattle potency test described by the OIE. A total of seventeen 3-month-old pigs were divided into four groups. Groups 1–3 (5 pigs each) were vaccinated intramuscularly with 1 dose (2 μg), 1/3 dose, and 1/9 dose of vaccine prepared from BEI-inactivated r-HN/3A_93–143_ vaccine antigen, respectively. Pigs in group 4 (2 pigs) were inoculated with MEM. At 28 dpv, all pigs were challenged intramuscularly in the neck with 1000 ID_50_ of the WT virus. The animals were then observed daily for the appearance of clinical signs of FMD infection. Control animals developed lesions on at least one foot, while protected animals did not show any clinical signs. The PD_50_ content of the vaccine was calculated based on the Spearman-Karber method.

### Differentiation ELISA assay

Serum samples collected from the animals infected either with r-HN or r-HN/3A_93–143_ at 28 dpi were examined for specific antibody to the targeted epitope using a blocking ELISA (bELISA) assay. Briefly, microplates (Costar 3590, Corning, NY, USA) were coated with FMDV-specific 2C polyclonal antibody produced in rabbits (100 μL/well) at a concentration of 1 μg/mL diluted in carbonate-bicarbonate buffer (pH 9.6), and incubated overnight at 4 °C. Prior to all steps, the plates were washed four times with PBST. During each subsequent step, the plates were incubated at 37 °C. Blocking buffer (PBS containing 1% gelatin) was added at 100 μL/well, and plates were incubated for 1 h at 37 °C. Purified FMDV 2C3AB protein [27] diluted in PBS was added at 1 μg/mL (100 μL/well), and the plates were incubated for 1 h at 37 °C. After incubation, serum samples in a 1:5 dilution in blocking buffer were added (100 μL/well), and incubated overnight at room temperature. Then, a volume of 100 μL of horseradish peroxidase (HRP)-conjugated FMDV 3A MAb (3A24) diluted in blocking buffer (1:200) was added to each well, and the plates were incubated for 1 h at 37 °C. Finally, the plates were incubated at room temperature for 10–15 min with TMB substrate (3, 3′, 5, 5′-tetramethylbenzidine), and stopped by the addition of 0.3 M sulfuric acid. The optical density (OD) was determined at 450 nm on an automated ELISA plate reader. The results were expressed as the percentage of inhibition using mean OD values of test sera as well as known FMDV-positive and -negative swine sera. The percent inhibition (PI) of samples was calculated as follows: PI = [(negative reference serum OD − test sample OD)/negative reference serum OD] × 100. The samples were considered positive if the PI values were greater than or equal to 46%.

## Results

### Generation of the marker FMDV

In order to introduce a negative marker into previously constructed plasmid pOFS, the overlap extension PCR was used to produce a targeted genome fragment. This fragment was digested by the appropriate restriction enzymes, and then cloned into pOFS plasmid to generate a modified FMDV full-length cDNA clone named pOFS/3A_93–143_. Sequence analysis of the mutant construct revealed that pOFS/3A_93–143_ contains the expected deletion and no other amino acid changes. Infectious virus was obtained by transfection of a linearized mutant full-length plasmid into BSR/T7 cells. The entire genomes of passage 4 of the marker virus and WT virus were sequenced to confirm that two viruses had no other mutations except for the presence of targeted deletion in the marker virus. The genetic stability of truncated-3A was also examined by nucleotide sequence analysis, and the results show that the engineered deletion in 3A was retained after up to 13 serial passages in BHK-21 cells.

### Characterization of the marker FMDV

**Figure 1:**
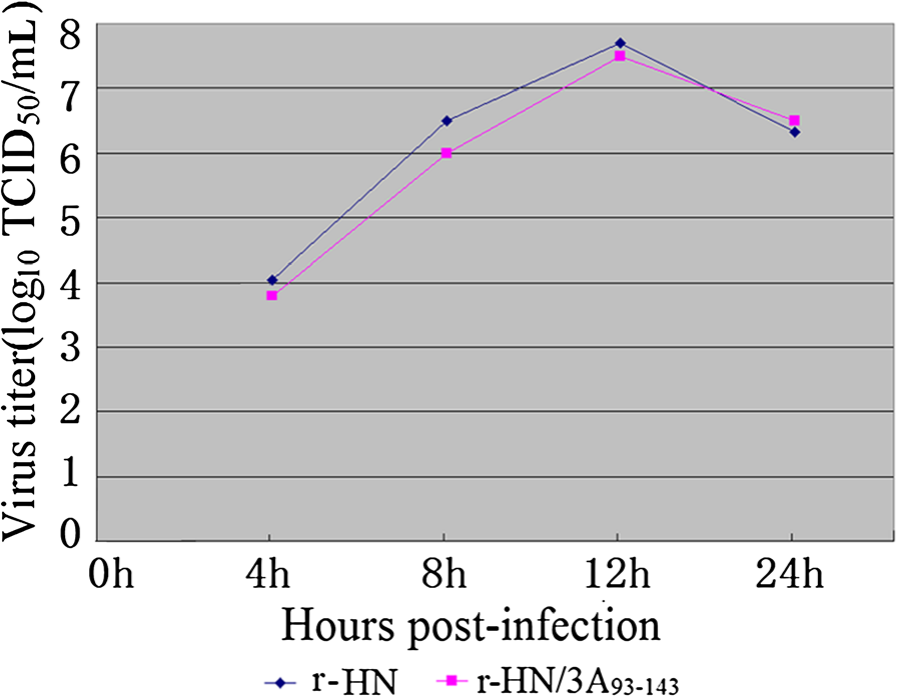
**Growth curves of r-HN and r-HN/3A**_**93–143 **_**in BHK-21 cells.** BHK-21 cells were infected either with r-HN or r-HN/3A_93–143_ at an MOI of 1. At 0, 4, 8, 12 and 24 h post-infection, cells and supernatants were harvested and the virus titers were determined by TCID_50_/mL on BHK-21 cells. The values of the viral titers represent the average obtained from triplicate experiments.

To investigate the possible effect of 3A length on virus growth, in vitro growth kinetics of the marker virus and WT virus were determined in BHK-21 monolayers at an MOI of 1. The single-step growth curves revealed no significant differences between these viruses, and they all reached the peak titers at 12 h post-infection (Figure 1). Additionally, the marker virus produced a plaque phenotype similar to that of the WT virus in BHK-21 cells (data not shown). These results suggest that the deletion of residues 93 to 143 in 3A does not affect virus replication in BHK-21 cells.

**Figure 2:**
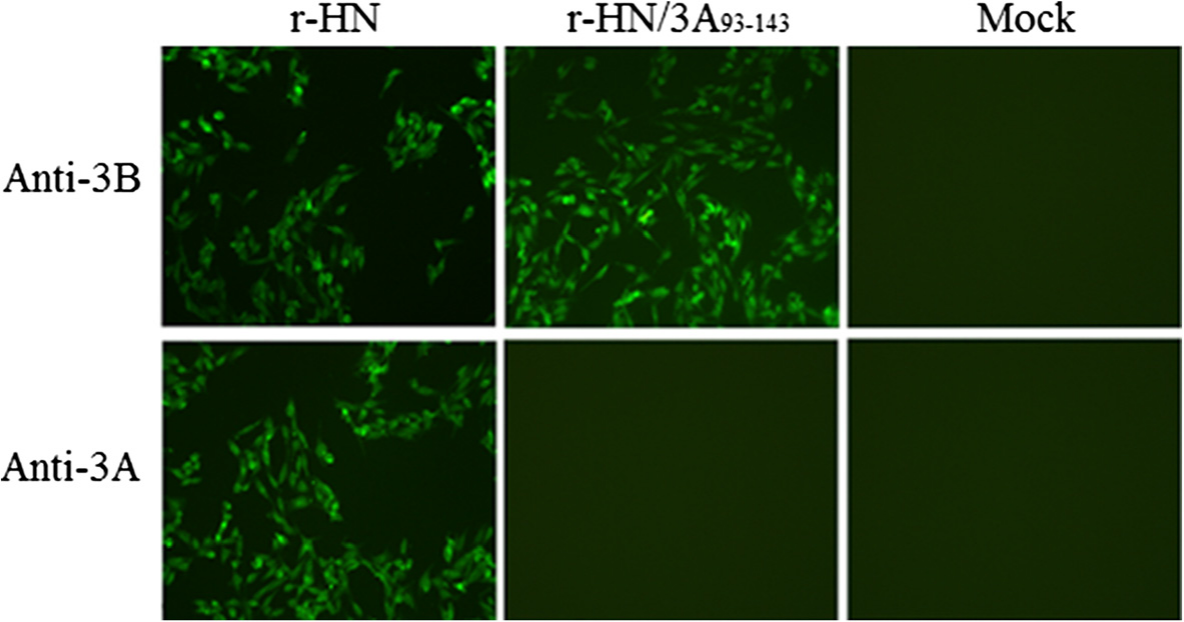
**Analysis of the marker epitope expression of the recombinant FMDV by immunofluorescence.** Confluent BHK-21 cells were either mock infected or infected with r-HN or r-HN/3A_93–143_ at an MOI of 1, incubated for 6 h, fixed and probed with anti-3A or anti-3B MAb, followed by incubation with fluorescein isothiocyanate (FITC)-conjugated secondary antibody. The cells were visualized under an Olympus BX40 fluorescence microscope. Magnification, ×10.

**Figure 3:**
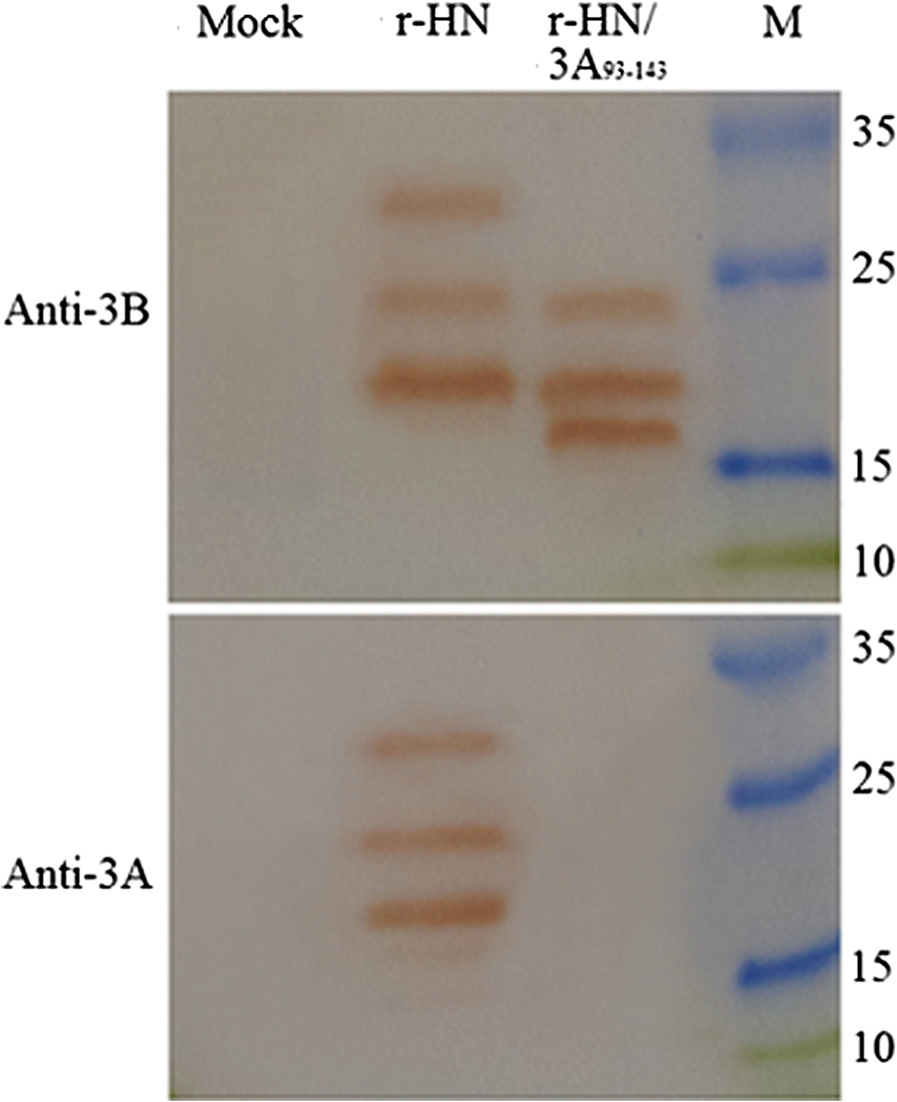
**Analysis of the marker epitope expression of the recombinant FMDV by western blotting.** BHK-21 cells were either mock infected or infected with r-HN or r-HN/3A_93–143_ at an MOI of 1, and incubated at 37 °C. Infected cell extracts were prepared at 12 h post-infection. Proteins were separated on a 12% SDS-PAGE, blotted, and probed with anti-3A or anti-3B MAb. Protein marker is indicated on the right.

To determine the antigenic properties and the size of the 3A protein, the r-HN or r-HN/3A_93–143_ -infected cells were examined by an immunofluorescent assay (Figure 2) and western blot analysis (Figure 3). As Figures 2 and 3 show, the WT virus reacted with both MAb 3A24 and 3B4B1, conversely, the marker virus reacted strongly with MAb 3B4B1, while completely lacking reactivity with MAb 3A24. Failure of MAb 3A24 to react with the marker virus shows that the deletion of residues 93 to 143 in 3A abolished the ability of the marker FMDV to be recognized by MAb 3A24 but not by MAb 3B4B1. In addition, Figure 3 shows that the mobility of all intermediates of the 3B protein (3B is too small to be readily observed by standard SDS-PAGE) of the marker virus was slightly faster than the corresponding intermediates of the WT virus, also indicating the marker virus is the right construction.

### Assessment of pathogenicity of the marker FMDV in swine

To evaluate the effect of the deletion in the 3A protein on the pathogenicity in pigs, we performed direct inoculation of r-HN or r-HN/3A_93–143_ in pigs. After inoculation, all animals produced an acute and synchronous disease. Clinical signs appeared by 2 to 4 dpi, and reached the maximum clinical score by 3 to 5 dpi (Table 2). Fever appeared at 2 to 4 dpi, and lasted for 3 to 5 dpi. Viremia was detected at 24 hpi, and reached a peak at 2 or 3 dpi, and lasted for 2 to 5 dpi. However, animals inoculated with the WT virus produced more vesicles beyond the injected sites and induced fever (Table 2) a day earlier than in the marker virus-inoculated animals. These results indicate that the marker virus can cause a symptomatic infection in pigs, but the clinical signs induced by the marker virus were milder than those induced by the WT virus. Tests on serum samples collected from inoculated animals at 7, 14, 21 and 28 dpi revealed that high FMDV-specific antibodies (≥ 1:128) were detectable at 7 dpi (data not shown), and reached higher levels at 14 dpi (≥ 512) (Table 2). All pigs developed antibodies to 3ABC at 28 dpi (Table 2), demonstrating that the viral replication occurred in inoculated animals. Furthermore, sequence analysis of each sample recovered from the pigs revealed that causative viruses were consistent with the inoculated viruses in their genome sequences, indicating that the targeted deletion remained stable during mutant virus growth in pigs.

**Table 2:** **Responses of swine directly inoculated with r-HN or r-HN/3A**_**93–143**_

**Virus**	**Pig**^**a**^	**Maximum viremia titre**^**b **^**(dpi)**	**Fever**^**c **^**(dpi)**	**Maximum clinical Score**^**d **^**(dpi)**	**Maximum LPBE-antibody**^**e **^**(dpi)**	**3ABC antibody**^**f **^**(dpi)**
r-HN	2408	7.23 (2)	Yes (2 and 3)	4/5 (3)	1:1024 (14)	+
r-HN	2411	7.7 (2)	Yes (2 to 5)	3/5 (4)	1:512 (14)	+
r-HN	2412	6.9 (3)	Yes (3 to 5)	4/5 (5)	1:720 (7)	+
r-HN/3A_93–143_	2413	7.1 (2)	Yes (3 to 5)	4/5 (4)	1:720 (14)	+
r-HN/3A_93–143_	2421	6.0 (3)	Yes (3 to 5)	2/5 (3)	1:512 (14)	+
r-HN/3A_93–143_	2422	7.3 (3)	Yes (4 to 5)	2/5 (5)	1:720 (7)	+

^a^Pigs were inoculated with 10^6^ TCID_50_ of r-HN or r-HN/3A_93–143_ by intradermal route.

^b^Values are expressed as the log_10_ RNA copies/mL. Dpi here indicates the day(s) post-inoculation that the virus peak was detected.

^c^Fever defined as rectal temperature > 40 °C. Dpi here indicates the days when fever was detected.

^d^Clinical scores were scored as previously described [24]. The maximum score is 5. Dpi here indicates the day after inoculation that the maximum score was reached.

^e^LPBE-antibody, liquid-phase blocking ELISA antibody. Dpi here indicates the day after inoculation that the maximum LPBE-antibodies were reached.

^f^3ABC antibody, the antibody to the nonstructural protein 3ABC of FMDV. Dpi here indicates four-weeks after inoculation that 3ABC antibodies were detected. +, positive, −, negative.

### Swine protection experiment

To test the protective potential of inactivated vaccines prepared from the WT and marker viruses in swine, we designed a swine vaccination and challenge study. As shown in Table 3, all vaccinated pigs developed high and similar levels of FMDV-specific antibodies at 28 dpv, however, in contrast, the unvaccinated controls did not produce detectable FMDV-specific antibody (Table 3). After challenge, the two unvaccinated controls developed fever and anorexia, followed by typical FMD lesions on feet, however, the animals vaccinated with vaccines did not show any clinical signs of FMD (Table 3), demonstrating that all animals vaccinated with the r-HN/3A_93–143_ and r-HN vaccines were completely protected from challenge with the WT virus. None of the animals employed in this study was positive for antibody to 3ABC at 28 dpv, however, the two unvaccinated controls developed significant antibody response against 3ABC at 21 days post challenge (Table 3), demonstrating that only the unvaccinated controls had become infected after challenge with the WT virus.

**Table 3:** **Responses of pigs to vaccination with r-HN or r-HN/3A**_**93–143 **_**chemically inactivated vaccines and r-HN challenge**

**Vaccine**^**a**^	**Pig**	**Protection**^**b**^	**LPBE-antibody**^**c **^**(dpi)**	**3ABC antibody**^**d **^**(dpi)**
r-HN/3A_93–143_	3501	yes	1:180	–
	3502	yes	1:512	–
	3503	yes	1:64	–
	3504	yes	1:128	–
r-HN	3505	yes	1:90	–
	3505	yes	1:256	–
	3507	yes	1:256	–
	3508	yes	1:32	–
Negative controls	3509	no	1:4	+
	3510	no	1:4	+

^a^Binary ethyleimine-inactivated antigen vaccine, 2 μg/2 mL in one dose.

^b^Pigs were challenged by intramuscular inoculation of 1000 ID_50_ of r-HN.

^c^LPBE-antibody, liquid-phase blocking ELISA antibody. Dpi here indicates four-weeks after vaccination that LPBE-antibodies were detected.

^d^3ABC antibody, the antibody to the nonstructural protein 3ABC of FMDV. Dpi here indicates three-weeks after challenge that 3ABC antibodies were detected. +, positive, −, negative.

### Potency of the marker vaccine in swine

The potency of marker vaccine was estimated in vaccinated pigs. As expected, the unvaccinted controls developed severe symptoms of FMD, including fever, anorexia and vesicular lesions on the feet. In contrast, 13/15 vaccinated animals did not show any clinical signs of FMD during the course of the experiment, whereas two animals (animals 1527 and 1532) vaccinated with 1/9 dose of marker vaccine developed delayed and slight FMD clinical signs compared with the unvaccinated controls (Table 4), indicating that 13/15 animals vaccinated with different doses of marker vaccine were protected from challenge with the WT virus. According to the Spearman-Karber method, the potency of negative marker vaccine reached 10 PD_50_ per dose, indicating that the marker virus is a potential vaccine candidate.

**Table 4:** **The result of the PD**_**50 **_**test of the r-HN/3A**_**93–143 **_**vaccine**

**Vaccine dose**^**a**^	**Pig**	**Protection**^**b**^	**LPBE-antibody**^**c **^**(dpi)**	**3ABC antibody**^**d **^**(dpi)**
1	1501	yes	1:512	–
	1503	yes	1:256	–
	1504	yes	1:128	–
	1528	yes	1:64	–
	1536	yes	1:256	–
1/3	1502	yes	1:128	–
	1506	yes	1:32	–
	1529	yes	1:256	–
	1534	yes	1:64	–
	1533	yes	1:256	–
1/9	1505	yes	1:64	–
	1507	yes	1:128	–
	1527	no	< 1:6	+
	1532	no	< 1:6	+
	1535	yes	1:512	–
control	026	no	< 1:6	+
	030	no	< 1:6	+

^a^2 μg per dose of binary ethyleimine-inactivated antigen vaccine.

^b^Pigs were challenged by intramuscular inoculation of 1000 ID_50_ of r-HN.

^c^LPBE-antibody, liquid-phase blocking ELISA antibody. Dpi here indicates four-weeks after vaccination that LPBE-antibodies were detected.

^d^3ABC antibody, the antibody to the nonstructural protein 3ABC of FMDV. dpi here indicates three-weeks after challenge that 3ABC antibodies were detected. +, positive, −, negative.

### Differential antibody responses against 3A in animals inoculated with negative marker virus

**Figure 4:**
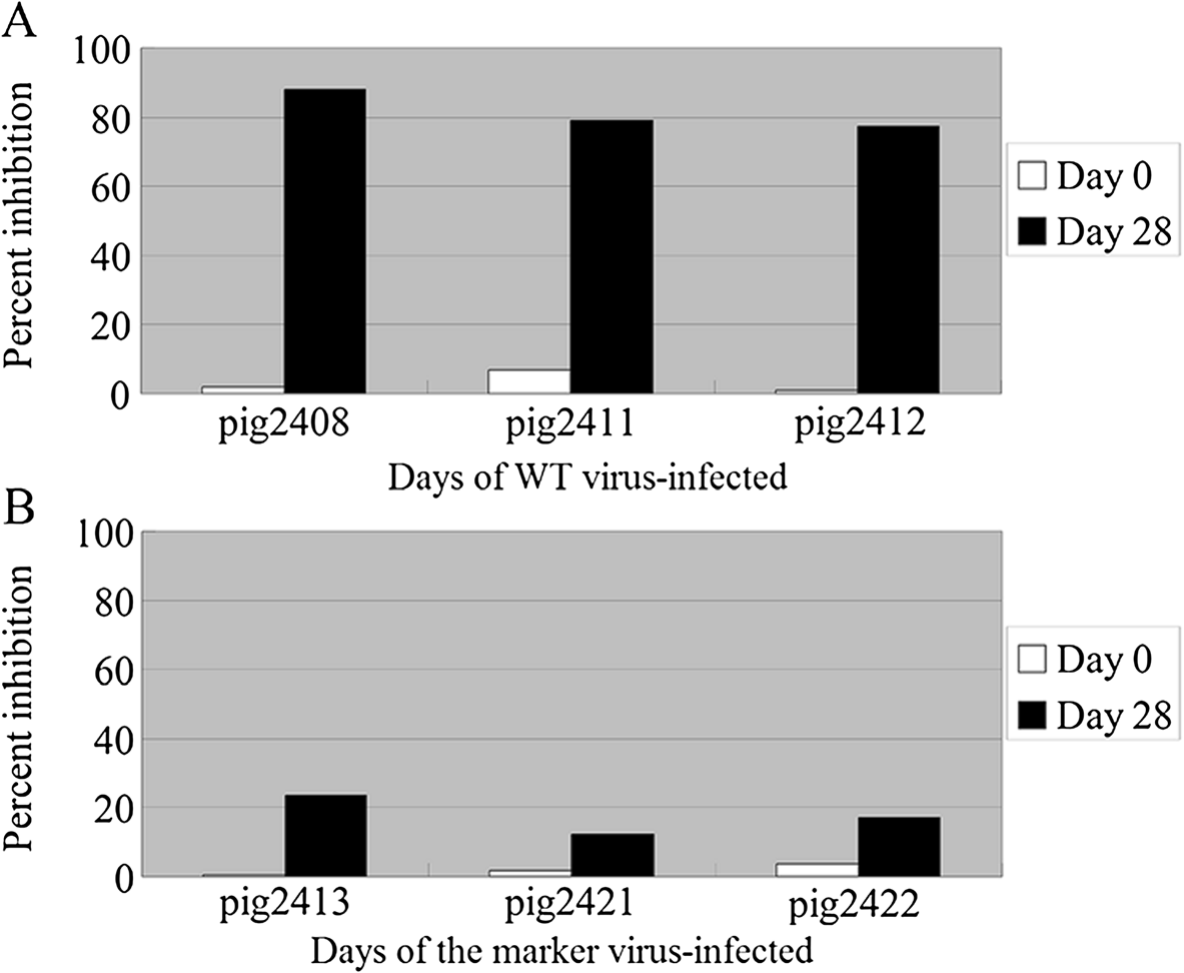
**bELISA. (A)** Differential antibody response in animals infected with WT virus using MAb 3A24 raised against 3A. **(B)** Differential antibody response in animals infected with the marker virus using MAb 3A24 raised against 3A. Samples were collected before inoculation and at 28 dpi.

To determine whether animals inoculated with the WT virus and marker virus developed antibody responses to the epitope of “AEKNPLE” of 3A protein, sera obtained from pre-inoculated (0 dpv) or convalescent-phase (28 dpv) animals were detected by a bELISA developed using MAb 3A24. We observed that the sera from all pre-inoculated pigs and pigs inoculated with the marker virus showed little inhibition (IP < 25%) of MAb 3A24 binding to 2C3AB protein at 28 dpi; in contrast, the sera from animals inoculated with the WT virus exhibited significant inhibition (IP > 70%) of MAb 3A24 binding at 28 dpi (Figure 4). In other words, the pigs infected with this marker virus did not develop a measurable antibody against the targeted epitope, while the pigs inoculated with the WT virus induced a high-level antibody to the corresponding epitope at 28 dpi. This indicates that the deletion of residues 93 to 143 in 3A abolished the binding ability of MAb 3A24 to the truncated 3A protein. These serological results show that a bELISA developed using MAb 3A24 allows for differentiation of animals infected with the wild type virus from those inoculated with the marker virus.

## Discussion

The use of marker vaccines have become very attractive and even mandatory in campaigns aiming toward the control or eradication of economically devastating animal diseases. To date, several marker vaccines including DNA vaccines [28,29], subunit vaccines [30,31], peptide vaccines [32,33] as well as genetically modified virus vaccines [34-36]) have been developed using various strategies. For these vaccines, the DIVA principle is based on a vaccine producing an antibody response that is different from the antibody responses produced by the WT virus. However, epidemiological and regulatory considerations suggested that a DIVA vaccine should be developed by deletion of an immunodominant epitope or certain viral protein to create a negative marker [24,34,35]. Herpesvirus negative marker vaccines were first developed in 1980, and their use has contributed to disease control and eradication in some countries [11,37,38]. Following the successes of these vaccines, negative marker vaccines of some animal diseases have been developed by identifying and selecting non-essential genes [35,39-42], and some of them have successfully been used to control disease outbreaks [9,10].

The studies of other viral negative marker vaccines provide insight into the development of a new marker vaccine against FMD. Recently, an FMDV negative marker vaccine was developed by deleting partial VP1 G–H loop [43], and the subsequent result demonstrated that this FMD negative marker vaccine can fully protect cattle from experimental challenge and meet the DIVA purpose [35]. However, not only the G-H loop of the VP1 protein, but also other antigenic sites of the capsid proteins are responsible for the complete immunogenicity of the FMDV vaccine [44,45]. Therefore, it is recommended that the negative marker vaccine should be designed by deletion or modification of some epitopes in NSPs [46]. More recently, FMDV marker vaccines featuring the deletion of the leader protein have been produced by modification or deletion of specific epitopes in NSP 3D and/or 3B [47]. These attenuated, antigenically marked vaccines enable serological differentiation infected from vaccinated animals in conjunction with a companion diagnostic test. However, a major disadvantage of live-attenuated vaccines is that the viruses were too attenuated to induce consistent and protective immune responses against virus infection [48,49]. Owing to these problems, an improved marker vaccine needs to be developed to assist FMD control.

Sequence alignment and our studies (data not yet published) showed that the epitope of residues 99 to 105 of the 3A protein is well-conserved and immunodominant among different serotypes of FMDV. Previous reports have shown that the FMDV 3A protein can tolerate some deletions (residues 93 to 144, 91 to 104) without affecting the replicating abilities in the BHK-21 cell [50,51]. More recently, FMDV with a deletion of residues 87 to 106 in 3A can also be recovered from full-length infectious cDNA clones [52]. Although the viruses featuring these deletions in 3A have been successfully rescued, the potential DIVA capabilities of these viruses have not been investigated. Therefore, in the present study, we constructed a marker FMDV containing the deletion of residues 93 to 143 in the 3A protein. We observed that the marker virus had similar growth kinetics and plaque morphology with the WT virus in the BHK-21 cell and was genetically stable after 13 serial passages in BHK-21 cells, indicating its potential advantages for production of the vaccine against FMDV.

In this study, a negative marker was introduced into FMDV by deletion of residues 93 to 143 in the 3A protein, which contains an immunodominant B-cell epitope of residues 99 to 105 (AEKNPLE). The results of the immunofluorescent assay and western blot analysis showed that MAb 3A24 had high reactivity with the WT virus, but failed to react with the marker virus. Based on the property of MAb 3A24, we developed a bELISA with this MAb to detect differential antibody response in animals infected with the WT virus and marker virus. As expected, pigs infected with this marker virus did not develop a measurable antibody against the targeted epitope by a developed blocking ELISA, but the pigs infected with the wild type virus produced a high-level antibody to the corresponding deleted epitope at 28 dpi, demonstrating that a developed bELISA could clearly differentiate animals infected with the WT virus from those inoculated with the marker virus. Therefore, the epitope “AEKNPLE” of 3A is a suitable target for the development of negative marker vaccine with DIVA capability.

Systematic vaccination programs with BEI-inactivated whole virus antigen used in conjunction with oil adjuvants have successfully reduced the number of FMD outbreaks in the enzootic areas of the world. In the current work, we compared the protective efficacy of the vaccine made from BEI-inactivated r-HN/3A_93–143_ or r-HN in pigs. The results demonstrate that a single vaccination with these vaccines protected all pigs from challenge with WT virus, and all vaccinated animals developed high and similar levels of FMDV-specific antibodies compared with the unvaccinated controls, indicating that the marker virus has good immunogenicity as the WT virus. In addition, our study shows that the potency of the marker vaccine could get 10 PD_50_ per dose for pigs. In this study, animals vaccinated with a single-dose r-HN/3A_93–143_ at 28 dpv did not produce a significant antibody response to NSP 3ABC and the targeted epitope (data not shown), in contrast, animals infected with the marker virus all developed antibody responses to NSP 3ABC that were unable to block the binding of 3A MAb targeted against the marker epitope at four weeks following inoculation. These results demonstrate that this recombinant virus with a negative marker is a potential marker vaccine candidate, enabling serological discrimination between vaccinated and infected animals utilizing developed blocking ELISA.

Taken together, our study indicates that the deletion of the immunodominant epitope in the 3A protein of FMDV can be potentially useful as a negative marker for the development of the DIVA vaccine to help FMD control and serosurveillance.

## Competing interests

The authors declare that they have no competing interests.

## Authors’ contributions

PL, HY and ZLi conceived and designed all the experiments. ZLu participated in the overall planning of the animal experiment, as well as analyzed the data. XB constructed the mutant FMDV cDNA clone and rescued the recombinant FMDV. DL and PS carried out the animal experiments. HB performed rRT-PCR and analyzed the data. YF and YCa detected animal sera. YCh and BX participated in the overall planning of the experiment. PL wrote the manuscript. All authors commented and approved the final manuscript.

## References

[B1] Samuel AR Knowles NJ Foot-and-mouth disease type O viruses exhibit genetically and geographically distinct evolutionary lineages (topotypes) J Gen Virol 2001 82 609 621 11172103 10.1099/0022-1317-82-3-609

[B2] Belsham GJ Distinctive features of foot-and-mouth disease virus, a member of the picornavirus family: aspects of virus protein synthesis, protein processing and structure Prog Biophys Mol Biol 1993 60 241 260 10.1016/0079-6107(93)90016-D 8396787 PMC7173301

[B3] Porter AG Picornavirus nonstructural proteins: emerging roles in virus replication and inhibition of host cell functions J Virol 1993 67 6917 6921 8230412 10.1128/jvi.67.12.6917-6921.1993 PMC238148

[B4] Doel TR FMD vaccines Virus Res 2003 91 81 99 10.1016/S0168-1702(02)00261-7 12527439

[B5] Mackay DK Forsyth MA Davies PR Salt JS Antibody to the non-structural proteins of foot-and-mouth disease virus in vaccinated animals exposed to infection Vet Q 1998 20 Supp 2 S9 S11 9652054 10.1080/01652176.1998.9694953

[B6] Sorensen KJ Madsen KG Madsen ES Salt JS Nqindi J Mackay DKJ Differentiation of infection from vaccination in foot-and-mouth disease by the detection of antibodies to the non-structural proteins 3D 3AB and 3ABC in ELISA using antigens expressed in baculovirus Arch Virol 1998 143 1461 1476 10.1007/s007050050390 9739326

[B7] Robiolo B Seki C Fondevilla N Grigera P Scodeller E Periolo O La Torre J Mattion N Analysis of the immune response to FMDV structural and non-structural proteins in cattle in Argentina by the combined use of liquid phase and 3ABC-ELISA tests Vaccine 2006 24 997 1008 10.1016/j.vaccine.2005.08.071 16171905

[B8] Armstrong RM Cox SJ Aggarwal N Mackay DJ Davies PR Hamblin PA Dani P Barnett PV Paton DJ Detection of antibody to the foot-and-mouth disease virus (FMDV) non-structural polyprotein 3ABC in sheep by ELISA J Virol Methods 2005 125 153 163 10.1016/j.jviromet.2005.01.012 15794985

[B9] Cattoli G Terregino C Brasola V Rodriguez JF Capua I Development and preliminary validation of an ad hoc N1-N3 discriminatory test for the control of avian influenza in Italy Avian Dis 2003 47 1060 1062 10.1637/0005-2086-47.s3.1060 14575111

[B10] Lee CW Senne DA Suarez DL Generation of reassortant influenza vaccines by reverse genetics that allows utilization of a DIVA (Differentiating Infected from Vaccinated Animals) strategy for the control of avian influenza Vaccine 2004 22 3175 3181 10.1016/j.vaccine.2004.01.055 15297071

[B11] Van Oirschot JT Kaashoek MJ Rijsewijk FA Stegeman JA The use of marker vaccines in eradication of herpesviruses J Biotechnol 1996 44 75 81 10.1016/0168-1656(95)00129-8 8717389

[B12] Peeters BP de Leeuw OS Verstegen I Koch G Gielkens AL Generation of a recombinant chimeric Newcastle disease virus vaccine that allows serological differentiation between vaccinated and infected animals Vaccine 2001 19 1616 1627 10.1016/S0264-410X(00)00419-9 11166884

[B13] Bird BH Albarino CG Hartman AL Erickson BR Ksiazek TG Nichol ST Rift valley fever virus lacking the NSs and NSm genes is highly attenuated, confers protective immunity from virulent virus challenge, and allows for differential identification of infected and vaccinated animals J Virol 2008 82 2681 2691 10.1128/JVI.02501-07 18199647 PMC2258974

[B14] Li P Bai X Sun P Li D Lu Z Cao Y Fu Y Bao H Chen Y Xie B Liu Z Evaluation of a genetically modified foot-and-mouth disease virus vaccine candidate generated by reverse genetics BMC Vet Res 2012 8 57 10.1186/1746-6148-8-57 22591597 PMC3488552

[B15] Buchholz UJ Finke S Conzelmann KK Generation of bovine respiratory syncytial virus (BRSV) from cDNA: BRSV NS2 is not essential for virus replication in tissue culture, and the human RSV leader region acts as a functional BRSV genome promoter J Virol 1999 73 251 259 9847328 10.1128/jvi.73.1.251-259.1999 PMC103829

[B16] Li P Bai X Cao Y Han C Lu Z Sun P Yin H Liu Z Expression and stability of foreign epitopes introduced into 3A nonstructural protein of foot-and-mouth disease virus PLoS One 2012 7 e41486 10.1371/journal.pone.0041486 22848509 PMC3407237

[B17] Han J Liu G Wang Y Faaberg KS Identification of nonessential regions of the nsp2 replicase protein of porcine reproductive and respiratory syndrome virus strain VR-2332 for replication in cell culture J Virol 2007 81 9878 9890 10.1128/JVI.00562-07 17522233 PMC2045381

[B18] Rieder E Bunch T Brown F Mason PW Genetically engineered foot-and-mouth disease viruses with poly(C) tracts of two nucleotides are virulent in mice J Virol 1993 67 5139 5145 8394441 10.1128/jvi.67.9.5139-5145.1993 PMC237911

[B19] Beard CW Mason PW Genetic determinants of altered virulence of Taiwanese foot-and-mouth disease virus J Virol 2000 74 987 991 10.1128/JVI.74.2.987-991.2000 10623761 PMC111619

[B20] Rieder E Henry T Duque H Baxt B Analysis of a foot-and-mouth disease virus type A24 isolate containing an sgd receptor recognition site in vitro and its pathogenesis in cattle J Virol 2005 79 12989 12998 10.1128/JVI.79.20.12989-12998.2005 16189001 PMC1235811

[B21] Oem JK Yeh MT McKenna TS Hayes JR Rieder E Giuffre AC Robida JM Lee KN Cho IS Fang X Joo YS Park JH Pathogenic characteristics of the Korean 2002 isolate of foot-and-mouth disease virus serotype O in pigs and cattle J Comp Pathol 2008 138 204 214 10.1016/j.jcpa.2008.01.007 18384806

[B22] Shao JJ Wong CK Lin T Lee SK Cong GZ Sin FW Du JZ Gao SD Liu XT Cai XP Xie Y Chang HY Liu JX Promising multiple-epitope recombinant vaccine against foot-and-mouth disease virus type o in swine Clin Vaccine Immunol 2011 18 143 149 10.1128/CVI.00236-10 21084463 PMC3019777

[B23] Lu Z Cao Y Guo J Qi S Li D Zhang Q Ma J Chang H Liu Z Liu X Xie Q Development and validation of a 3ABC indirect ELISA for differentiation of foot-and-mouth disease virus infected from vaccinated animals Vet Microbiol 2007 125 157 169 10.1016/j.vetmic.2007.05.017 17601688

[B24] Fowler VL Bashiruddin JB Maree FF Mutowembwa P Bankowski B Gibson D Cox S Knowles N Barnett PV Foot-and-mouth disease marker vaccine: cattle protection with a partial VP1 G-H loop deleted virus antigen Vaccine 2011 29 8405 8411 10.1016/j.vaccine.2011.08.035 21856354

[B25] Li D Lu ZJ Xie BX Sun P Chen YL Fu YF Liu ZX Alternative way to test the efficacy of swine FMD vaccines: measurement of pigs median infected dose (PID50) and regulation of live virus challenge dose Viral J 2010 7 215 10.1186/1743-422X-7-215 PMC2944167 20822547

[B26] Cao Y Lu Z Li Y Sun P Li D Li P Bai X Fu Y Bao H Zhou C Xie B Chen Y Liu Z Poly(I:C) combined with multi-epitope protein vaccine completely protects against virulent foot-and-mouth disease virus challenge in pigs Antiviral Res 2013 97 145 153 10.1016/j.antiviral.2012.11.009 23219974

[B27] Lu Z Zhang X Fu Y Cao Y Tian M Sun P Li D Liu Z Xie Q Expression of the major epitope regions of 2C integrated with the 3AB non-structural protein of foot-and-mouth disease virus and its potential for differentiating infected from vaccinated animals J Virol Methods 2010 170 128 133 10.1016/j.jviromet.2010.09.016 20863858

[B28] Nobiron I Thompson I Brownlie J Collins ME Cytokine adjuvancy of BVDV DNA vaccine enhances both humoral and cellular immune responses in mice Vaccine 2001 19 4226 4235 10.1016/S0264-410X(01)00157-8 11457549

[B29] Wienhold D Armengol E Marquardt A Marquardt C Büttner M Saalmüller A Pfaff E Immunomodulatory effect of plasmids co-expressing cytokines in classical swine fever virus subunit gp55/E2-DNA vaccination Vet Res 2005 36 571 587 10.1051/vetres:2005019 15955282

[B30] Moormann RJ Bouma A Kramps JA Terpstra C De Smit HJ Development of a classical swine fever subunit marker vaccine and companion diagnostic test Vet Microbiol 2000 73 209 219 10.1016/S0378-1135(00)00146-2 10785329

[B31] Ahrens U Kaden V Drexler C Visser N Efficacy of the classical swine fever (CSF) marker vaccine Porcilis Pesti in pregnant sows Vet Microbiol 2000 77 83 87 10.1016/S0378-1135(00)00265-0 11042402

[B32] Dong XN Chen Y Wu Y Chen YH Candidate multi-peptide-vaccine against classical swine fever virus induced potent immunity with serological marker Vaccine 2005 23 3630 3633 10.1016/j.vaccine.2005.02.008 15882522

[B33] Hollinger FB Dreesman GR Sparrow J Melnick JL Synthetic peptide vaccine for hepatitis Dev Biol Stand 1983 54 113 116 6228451

[B34] Fang Y Christopher-Hennings J Brown E Liu H Chen Z Lawson SR Breen R Clement T Gao X Bao J Knudsen D Daly R Nelson E Development of genetic markers in the non-structural protein 2 region of a US type 1 porcine reproductive and respiratory syndrome virus: implications for future recombinant marker vaccine development J Gen Virol 2008 89 3086 3096 10.1099/vir.0.2008/003426-0 19008397

[B35] Buczkowski H Parida S Bailey D Barrett T Banyard AC A novel approach to generating morbillivirus vaccines: negatively marking the rinderpest vaccine Vaccine 2012 30 1927 1935 10.1016/j.vaccine.2012.01.029 22265946

[B36] Parida S Mahapatra M Kumar S Das SC Baron MD Anderson J Barrett T Rescue of a chimeric rinderpest virus with the nucleocapsid protein derived from peste-des-petits-ruminants virus: use as a marker vaccine J Gen Virol 2007 88 2019 2027 10.1099/vir.0.82913-0 17554036 PMC2885620

[B37] Moormann RJ de Rover T Briaire J Peeters BP Gielkens AL van Oirschot JT Inactivation of the thymidine kinase gene of a gI deletion mutant of pseudorabies virus generates a safe but still highly immunogenic vaccine strain J Gen Virol 1990 71 1591 1595 10.1099/0022-1317-71-7-1591 2165138

[B38] Kaashoek MJ Moerman A Madic J Rijsewijk FA Quak J Gielkens AL van Oirschot JT A conventionally attenuated glycoprotein E-negative strain of bovine herpesvirus type 1 is an efficacious and safe vaccine Vaccine 1994 12 439 444 10.1016/0264-410X(94)90122-8 8023552

[B39] de Lima M Kwon B Ansari IH Pattnaik AK Flores EF Osorio FA Development of a porcine reproductive and respiratory syndrome virus differentiable (DIVA) strain through deletion of specific immunodominant epitopes Vaccine 2008 26 3594 3600 10.1016/j.vaccine.2008.04.078 18538899

[B40] Castillo-Olivares J Wieringa R Bakonyi T de Vries AAF Davis-Poynter NJ Rottier PJM Generation of a candidate live marker vaccine for equine arteritis virus by deletion of the major virus neutralization domain J Virol 2003 15 8470 8480 12857916 10.1128/JVI.77.15.8470-8480.2003 PMC165223

[B41] Kortekaas J Ketelaar J Vloet RPM Loeffen WL Protective efficacy of a Classical swine fever virus C-strain deletion mutant and ability to differentiate infected from vaccinated animals Vet Microbiol 2011 147 11 18 10.1016/j.vetmic.2010.05.038 20541334

[B42] Wang L Suarez DL Pantin-Jackwood M Mibayashi M Garc´ıa-Sastre A Saif YM Lee CW Characterization of influenza virus variants with different sizes of the non-structural (NS) genes and their potential as a live influenza vaccine in poultry Vaccine 2008 26 3580 3586 10.1016/j.vaccine.2008.05.001 18539366 PMC2785844

[B43] Fowler VL Knowles NJ Paton DJ Barnett PV Marker vaccine potential of a foot-and-mouth disease virus with a partial VP1 G-H loop deletion Vaccine 2010 28 3428 3434 10.1016/j.vaccine.2010.02.074 20199761

[B44] Grubman MJ Baxt B Foot-and-mouth disease Clin Microbiol Rev 2004 17 465 493 10.1128/CMR.17.2.465-493.2004 15084510 PMC387408

[B45] Mateu MG Camarero JA Giralt E Andreu D Domingo E Direct evaluation of the immunodominance of a major antigenic site of foot-and-mouth disease virus in a natural host Virology 1995 206 298 306 10.1016/S0042-6822(95)80045-X 7831785

[B46] Uddowla S Hollister J Pacheco JM Rodriguez LL Rieder E A safe foot-and-mouth disease vaccine platform with two negative markers for differentiating infected from vaccinated animals J Virol 2012 86 11675 11685 10.1128/JVI.01254-12 22915802 PMC3486329

[B47] Rodriguez LL Gay CG Development of vaccines toward the global control and eradication of foot-andmouth disease Expert Rev Vaccines 2011 10 377 387 10.1586/erv.11.4 21434805

[B48] Martin WB Edwards LT A field trial in South Africa of an attenuated vaccine against foot-and-mouth disease Res Vet Sci 1965 6 196 201 14329718

[B49] Zhidkov SA Sergeev VA A study of the properties of attenuated cold variant of type O foot-and-mouth disease virus Veterinariia 1969 10 29 31 (in Russian) 4314613

[B50] Pacheco JM Henry TM O’Donnell VK Gregory JB Mason PW Role of nonstructural proteins 3A and 3B in host range and pathogenicity of foot-and-mouth disease virus J Virol 2003 77 13017 13027 10.1128/JVI.77.24.13017-13027.2003 14645558 PMC296074

[B51] Li S Gao M Zhang R Song G Song J Liu D Cao Y Li T Ma B Liu X Wang J A mutant of Asia 1 serotype of Foot-and-mouth disease virus with the deletion of an important antigenic epitope in the 3A protein Can J Microbiol 2011 57 169 176 10.1139/W10-112 21358757

[B52] Pacheco JM Gladue DP Holinka LG Arzt J Bishop E Smoliga G Pauszek SJ Bracht AJ O’Donnell V Fernandez-Sainz I Fletcher P Piccone ME Rodriguez LL Borca MV A partial deletion in non-structural protein 3A can attenuate foot-and-mouth disease virus in cattle Virology 2013 446 260 267 10.1016/j.virol.2013.08.003 24074589

